# Augmented renal clearance in septic and traumatized patients with normal plasma creatinine concentrations: identifying at-risk patients

**DOI:** 10.1186/cc12544

**Published:** 2013-02-28

**Authors:** Andrew A Udy, Jason A Roberts, Andrew F Shorr, Robert J Boots, Jeffrey Lipman

**Affiliations:** 1 Burns, Trauma, and Critical Care Research Centre, University of Queensland, Royal Brisbane and Womens Hospital, Butterfield Street, Herston 4029, Australia; 2 Department of Intensive Care Medicine, Royal Brisbane and Womens Hospital, Butterfield Street, Herston 4029, Australia; 3 Pharmacy Department, Royal Brisbane and Womens Hospital, Butterfield Street, Herston 4029, Australia; 4 Pulmonary and Critical Care Medicine, Washington Hospital Center, 110 Irving Street NW, Washington DC 20010, USA

## Abstract

**Introduction:**

Improved methods to optimize drug dosing in the critically ill are urgently needed. Traditional prescribing culture involves recognition of factors that mandate dose reduction (such as renal impairment), although optimizing drug exposure, through more frequent or augmented dosing, represents an evolving strategy. Elevated creatinine clearance (CL_CR_) has been associated with sub-therapeutic antibacterial concentrations in the critically ill, a concept termed augmented renal clearance (ARC). We aimed to determine the prevalence of ARC in a cohort of septic and traumatized critically ill patients, while also examining demographic, physiological and illness severity characteristics that may help identify this phenomenon.

**Methods:**

This prospective observational study was performed in a 30-bed tertiary level, university affiliated, adult intensive care unit. Consecutive traumatized and septic critically ill patients, receiving antibacterial therapy, with a plasma creatinine concentration ≤110 μmol/L, were eligible for enrolment. Pulse contour analysis (Vigileo / Flo Trac^® ^system, Edwards Lifesciences, Irvine, CA, USA), was used to provide continuous cardiac index (CI) assessment over a single six-hour dosing interval. Urinary CL_CR _measures were obtained concurrently.

**Results:**

Seventy-one patients contributed data (sepsis n = 43, multi-trauma n = 28). Overall, 57.7% of the cohort manifested ARC, although there was a greater prevalence in trauma (85.7% versus 39.5%, *P * <0.001). In all patients, a weak correlation was noted between CI and CL_CR _(r = 0.346, *P *= 0.003). This was mostly driven by septic patients (r = 0.508, *P *= 0.001), as no correlation (r = -0.012, *P *= 0.951) was identified in trauma. Those manifesting ARC were younger (*P* <0.001), male (*P *= 0.012), with lower acute physiology and chronic health evaluation (APACHE) II (*P*= 0.008) and modified sequential organ failure assessment (SOFA) scores (*P *= 0.013), and higher cardiac indices (*P *= 0.013). In multivariate analysis, age ≤50 years, trauma, and a modified SOFA score ≤4, were identified as significant risk factors. These had greater utility in predicting ARC, compared with CI assessment alone.

**Conclusions:**

Diagnosis, illness severity and age, are likely to significantly influence renal drug elimination in the critically ill, and must be regularly considered in future study design and daily prescribing practice.

See related commentary by De Waele and Carlier, http://ccforum.com/content/17/2/130

## Introduction

Accurate pharmaceutical prescription remains uniquely challenging in the critically ill. Many dosing schedules are simply extrapolated from data derived from healthy volunteers or ambulatory patients, without consideration of the pathophysiology [1] or clinical heterogeneity, often encountered in this setting. Capillary leak, fluid loading, decreased protein binding, use of vasoactive medications and altered excretory organ function, will significantly distort the 'normal' pharmacokinetic (PK) profile of many agents [2]. Most concerning is the potential effects on antibacterial drug exposure, given the wealth of data demonstrating improved outcomes with early appropriate therapy [3-5]. Although infrequently considered, such issues may not only confound the successful individual use of many pharmaceuticals, but also the planning, methodology and interpretation of clinical trials in this population [6].

A key PK variable of interest is drug clearance (CL), with previous data demonstrating notably elevated values in subsets of critically ill patients [7]. This phenomenon has recently been termed augmented renal clearance (ARC) [8] and may significantly impact the successful application of many renally eliminated agents by promoting sub-therapeutic drug exposure [8,9]. Although specific data concerning drug CL in critical illness remains sparse, elevated urinary creatinine clearance (CL_CR_), as a marker of ARC, has been documented in sepsis [10], ventilator associated pneumonia [11], traumatic brain injury [12], burns [13], multi-trauma [14] and post-operatively [15]. Furthermore, elevated CL_CR _has been closely linked with sub-therapeutic β-lactam antibacterial trough concentrations [16,17] in addition to being significantly correlated with renal drug elimination [7].

Identification of patients manifesting ARC remains clinically challenging, principally as many agents (most notably antibacterials) manifest 'silent' pharmacodynamic indices, making under-dosing substantially less visible [18]. Although various mathematical estimates of glomerular filtration are widely applied [19,20], each was primarily designed for use outside of the ICU, making application in this setting flawed [21,22] and of little value in guiding therapy. While a measured CL_CR _has greater utility [23], a defined urinary collection period is required, thereby limiting application to initial dose selection. Improved methods to identify patients with ARC using simple bedside assessment are urgently required.

The physiological alterations promoting ARC remain poorly understood. In large animal models of gram-negative sepsis, elevated cardiac index (CI), low systemic vascular resistance, and increased major organ blood flow have been demonstrated [24]. Application of aggressive fluid resuscitation [25] and vasopressor support [26] further augments this process, leading to substantial changes in renal function. Many parallels can be drawn with pregnancy, where similar cardiovascular changes are associated with augmented renal blood flow and glomerular filtration [27]. As such, in the absence of established acute kidney injury (AKI), the innate hemodynamic response to critical illness, coupled with common clinical interventions, may promote increased solute delivery to the kidneys and subsequent augmented renal elimination.

In this respect, assessment of CI offers a logical, pragmatic and physiologically sound method of rapidly assessing patients for the presence of ARC. To our knowledge, there has been little data reported on this application, representing a new, unique, indication for cardiovascular monitoring. Importantly, although CI assessment has historically employed invasive techniques (such as pulmonary artery catheterization), a variety of new devices are making continuous CI measurement accessible, feasible and safe [28]. The aims of this pilot prospective observational study were, therefore, to: a) describe the prevalence of ARC in a cohort of septic and traumatized critically ill patients receiving antibacterial therapy; b) correlate CL_CR _and CI in these patients; and c) examine demographic, physiological and illness severity characteristics that may help to identify patients manifesting ARC.

## Materials and methods

### Study population

Patients were enrolled consecutively as part of a wider open label study examining β-lactam antibacterial PK in critical illness, the methodology of which has been published elsewhere [29]. In brief, patients were eligible for enrolment if they were: a) 18 to 80 years of age; and b) receiving piperacillin-tazobactam for treatment of presumed or confirmed nosocomial infection, while manifesting a systemic inflammatory response syndrome (SIRS) [30], or were receiving cefazolin as prophylaxis following multi-trauma. This, therefore, represents a convenience sample of multi-trauma and septic critically ill patients admitted to our institution. This manuscript reports a separate, independent analysis, focusing on ARC. The study protocol was approved by our institutional human research ethics committee (HREC 2007/188) and informed consent was obtained from either the patient or their substitute decision maker in all cases.

### Study protocol

An in-depth physiological and PK investigation was performed over a single six-hour dosing interval following antibacterial infusion [29]. Pulse contour analysis, utilizing the Vigileo^® ^system (software version 1.10), connected to an existing intra-arterial catheter via a Flo Trac^® ^(Edwards Lifesciences, Irvine, CA, USA) sensor, was employed as the primary method of measuring CI. Demographic data including patient age, gender, body weight and height were inputted, following which the sensor was levelled to the phlebostatic axis and 'zeroed' to atmospheric pressure. The system provides continuous cardiac output data utilizing the heart rate and an index of stroke volume (obtained from the arterial pressure waveform), which is automatically averaged and updated. CI (L/min/m^2^) is then calculated as the cardiac output (L/min) divided by the body surface area (BSA) (m^2^). Three CI measurements were recorded at 0, 180, and 300 minutes, after which the mean value was calculated for use in subsequent analysis.

CL_CR _was measured as the primary method of determining kidney function. All urine was collected via an indwelling catheter over three two-hour time periods (0 to 120, 120 to 240, and 240 to 360 minutes, respectively), following which urinary volume and creatinine concentration were determined by laboratory analysis. Creatinine measurement in plasma and urine utilized automated analyzers employing a modified Jaffe (alkaline picrate) technique, representing an isotope dilution mass spectrometry (IDMS) traceable assay. Plasma creatinine concentrations measured on the day of investigation were used to calculate each CL_CR _(normalized to a BSA of 1.73 m^2^), after which the mean value was used in further analysis.

Additional data, including the requirement for mechanical ventilation, vasopressor support, modified sequential organ failure assessment (SOFA) score (excluding the neurological component) and 24-hour fluid balance, were also recorded on the day of drug administration. Admission acute physiology and chronic health evaluation (APACHE) II score, in addition to ICU and in-hospital clinical outcomes, were also recorded. Given that changes in cardiovascular physiology are unlikely to promote enhanced renal elimination in the setting of evolving AKI, patients with a plasma creatinine concentration greater than the upper limit of the reported reference range (>110 μ mol/L) were excluded from further analysis. ARC was defined as a CL_CR _≥130 ml/min/1.73 m^2^, given previous data demonstrating an association with sub-therapeutic β-lactam concentrations, when using standard doses [17].

### Statistics

Continuous data are presented as the mean (SD) or median [IQR]. Categorical data are presented as counts (%). Correlation was assessed by means of a scatter graph and Pearson correlation coefficient (r). Comparisons between groups utilized an Independent Student T-test or Mann-Whitney U test for continuous data, and a Chi-square or Fishers Exact test for categorical data, where analysis assumptions were met. A backward conditional logistic regression model was developed to describe risk factors for ARC in multivariate analysis. Covariates were identified if the associated *P*-value was <0.15 in univariate testing, and the Hosmer-Lemeshow statistic was used to assess goodness of fit. Receiver operator characteristic (ROC) curves were constructed to examine the accuracy of any variable to predict ARC. A *P*-value <0.05 was considered as indicating statistical significance, and all analyses were performed using SPSS version 19 (Chicago, IL, USA).

## Results

Eighty patients were enrolled in the open label PK study, fifty meeting the criteria for sepsis, and the remaining thirty admitted post multi-trauma. One patient was excluded from further analysis as no CI measurements were available, while a further eight patients were excluded due to a plasma creatinine concentration >110 μmol/L on the day of study. Laboratory, demographic, illness severity and outcome data for the remaining seventy-one patients (sepsis n = 43, multi-trauma n = 28) are presented in Table 1. As expected, young male patients dominated the multi-trauma group, although illness severity scores were similar between diagnostic categories. Data collection occurred a median of 1.60 [1.20 to 2.13] days post admission in the trauma sub-group, compared with 4.11 [1.68 to 6.83] days in sepsis (*P *<0.001). Crude ICU (4.2%) and in-hospital (8.5%) mortality were remarkably low.

**Table 1:** Laboratory, demographic and illness-severity data of all patients (n = 71).

Variable	All patients (number = 71)	Trauma (number = 28)	Sepsis (number = 43)	*P*-value^a^
Age, years, mean (SD)	42.4 (16.6)	36.4 (13.9)	46.3 (17.1)	0.013
Male gender, number (%)	45 (63.4)	23 (82.1)	22 (51.2)	0.008
BSA, m^2^, mean (SD)	1.98 (0.26)	2.01 (0.25)	1.96 (0.27)	0.415
APACHE II score, mean (SD)	17.9 (7.15)	16.1 (7.68)	19.0 (6.62)	0.096
Modified SOFA score, median [IQR]	3 [2-5]	3.5 [2-5]	3 [2-5]	0.659
Use of Vasopressors, number (%)	20 (28.2)	11 (39.3)	9 (20.9)	0.093
Mechanical ventilation, number (%)	66 (93.0)	26 (92.9)	40 (93.0)	1.000
24hour Fluid balance, ml, mean (SD)	656 (1,886)	1,209 (1903)	295 (1,806)	0.045
Plasma CR, μmol/L, mean (SD)	66.1 (18.1)	62.7 (13.2)	68.4 (20.5)	0.157
CI, L/min/m^2^, mean (SD)	4.20 (1.10)	4.30 (0.86)	4.13 (1.23)	0.507
CL_CR_, ml/min/1.73 m^2^, mean (SD)	135 (51.8)	166 (42.5)	114 (47.2)	<0.001
Augmented renal clearance, n (%)	41 (57.7)	24 (85.7)	17 (39.5)	< 0.001
ICU length of stay, days, mean (SD)	16.0 (11.1)	13.3 (10.2)	17.8 (11.4)	0.090
ICU mortality, number (%)	3 (4.20)	1 (3.6)	2 (4.7)	1.000
Hospital mortality, number (%)	6 (8.50)	3 (10.7)	3 (7.0)	0.674

^a^Comparison between sub-groups. APAHCE II, Acute Physiology and Chronic Health Evaluation; BSA, body surface area; CI, cardiac index; CL_CR_, creatinine clearance; CR, creatinine concentration; SOFA, Sequential Organ Failure Assessment.

**Figure 1:**
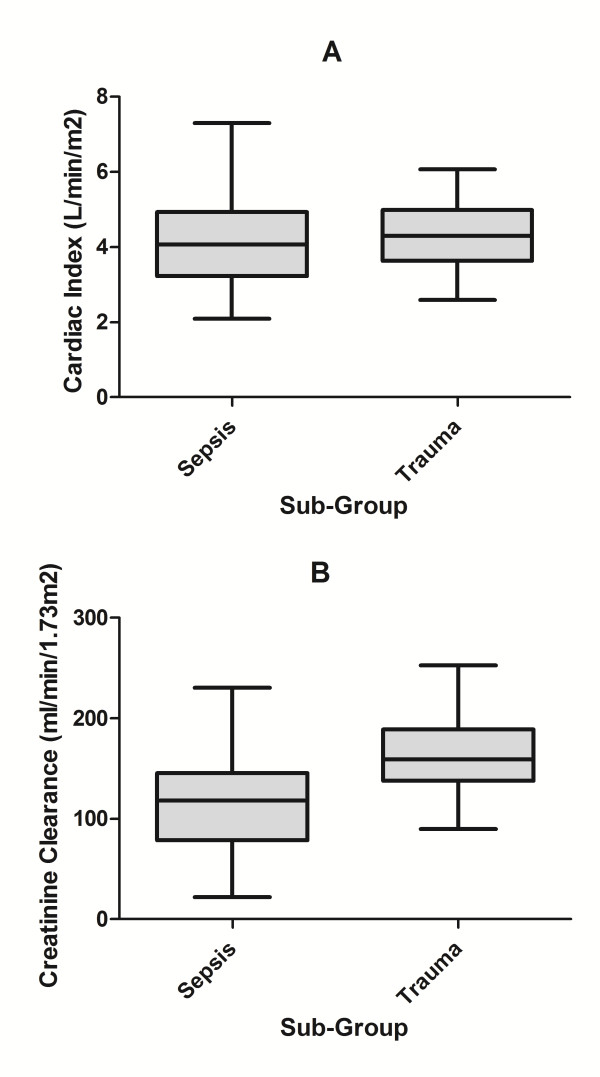
**Box plot of CI (L/min/m^2^) and CL_CR _(ml/min/1.73 m^2^) in trauma and septic patients**. Box plot (median, interquartile range, maximum and minimum) of cardiac index, L/min/m^2 ^(**A**) and creatinine clearance, ml/min/1.73 m^2 ^(**B**) in trauma (n = 28) and septic (n = 43) patients. Higher CL_CR _values were demonstrated in those admitted post trauma (*P *<0.001).

**Figure 2:**
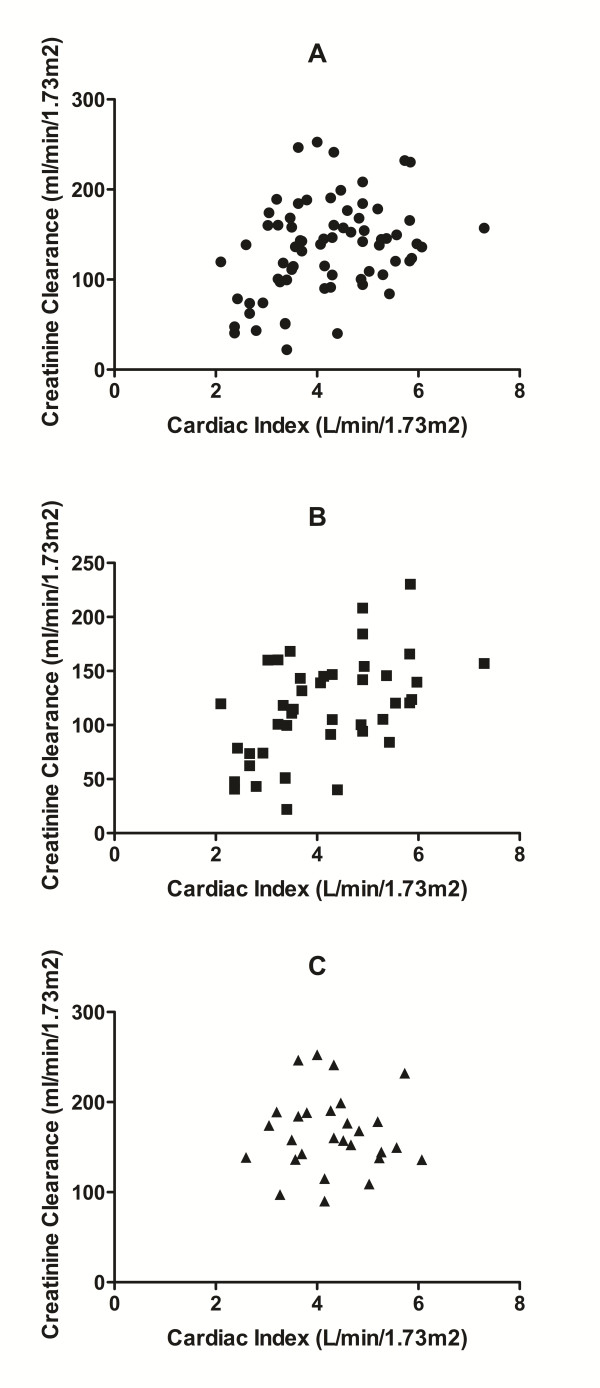
**Correlation of CI (L/min/m^2^) and CL_CR _(ml/min/1.73 m^2^)**. Scatter graphs of cardiac index (L/min/m^2^) and creatinine clearance (ml/min/1.73 m^2^) in all patients (**A**), septic patients (**B**) and trauma patients (**C**). The Pearson correlation coefficient (r) for all patients was r = 0.346 (*P *= 0.003), septic patients r = 0.508 (*P *= 0.001), and trauma patients r = -0.012 (*P *= 0.951).

Overall, 57.7% of the cohort manifested ARC (CL_CR _≥130 ml/min/1.73 m^2^), although higher CL_CR _values were noted in traumatized patients (166 (42.5) versus 114 (47.2) ml/min/1.73 m^2^, *P* <0.001), leading to a greater prevalence in this group (85.7% versus 39.5%, *P *<0.001). The range of CI and CL_CR _measures observed in each diagnostic sub-group are presented in Figure 1. In all patients (n = 71), a weak, statistically significant correlation was evident between CI and CL_CR _(r = 0.346, *P *= 0.003), although this was primarily due to the relationship observed in septic patients (r = 0.508, *P *= 0.001), as no correlation (r = -0.012, *P *= 0.951) was evident in trauma patients (see Figure 2).

**Figure 3:**
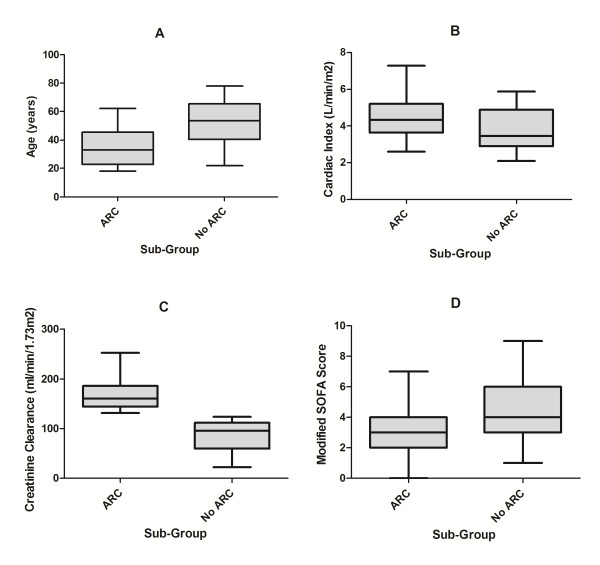
**Box plot of age (years), CI (L/min/m^2^), CL_CR _(ml/min/1.73 m^2^) and modified SOFA score in patients with and without augmented renal clearance**. Box plot (median, interquartile range, maximum and minimum) of age, years (**A**), cardiac index, L/min/m^2 ^(**B**), creatinine clearance, ml/min/1.73 m^2 ^(**C**) and modified SOFA score (**D**), in those with (n = 41) and without (n = 30) augmented renal clearance. Younger age (*P* <0.001), higher cardiac indices (*P *= 0.013) and lower modified SOFA scores (*P*= 0.013) were observed in those manifesting augmented renal clearance.

Differences in demographic, illness severity, physiological and laboratory data on the basis of ARC status are provided in Table 2. As illustrated, those manifesting ARC tended to be younger (*P* <0.001), male (*P *= 0.012), with lower APACHE II (*P *= 0.008) and modified SOFA scores (*P *= 0.013) and higher cardiac indices (P = 0.013). The range of values recorded for age, CI, CL_CR _and modified SOFA score are presented graphically in Figure 3.

**Table 2:** Demographic, diagnostic and treatment-related data in those with and without augmented renal clearance.

Variable	ARC (number = 41)	No ARC (number = 30)	*P*-value
Age, years, mean (SD)	34.1 (11.7)	53.7 (15.5)	<0.001
Male gender, number (%)	31 (75.6)	14 (46.7)	0.012
BSA, m^2^, mean (SD)	1.98 (0.25)	1.99 (0.28)	0.850
APACHE II score, mean (SD)	16.0 (6.33)	20.4 (7.49)	0.008
Modified SOFA score, median [IQR]	3 [2-4]	4 [3-6]	0.013
Use of vasopressors, number (%)	9 (22.0)	11 (36.7)	0.173
Mechanical ventilation, number (%)	39 (95.1)	27 (90.0)	0.644
24 hr Fluid balance, ml, mean (SD)	428 (2011)	967 (1684)	0.237
CI, L/min/m^2^, mean (SD)	4.47 (1.01)	3.80 (1.12)	0.013
CL_CR_, ml/min/1.73 m^2^, mean (SD)	170 (32.9)	86.8 (29.5)	<0.001
Category Trauma, number (%) Sepsis, number (%)	24 (58.5) 17 (41.5)	4 (13.3) 26 (86.7)	<0.001 <0.001

APACHE II, Acute Physiology and Chronic Health Evaluation score; BSA, body surface area; CI, cardiac index; CL_CR_, creatinine clearance; SOFA, Sequential Organ Failure Assessment.

Linear variables associated with ARC were then dichotomized to facilitate multivariate logistic regression. Cut-points were identified from visual inspection of the data (Figures 3). Specifically, age ≤50 years, CI ≥3.5 L/min/m^2 ^and modified SOFA score ≤4, along with gender and diagnostic sub-group, were entered as categorical variables into a backward conditional regression model. APACHE II scores were not included, as these are co-linear with age and SOFA, and poorly validated in trauma. This analysis identified age ≤50 years (adjusted odds ratio (OR) 28.6, 95% CI 4.4 to 187.2), trauma (adjusted OR 16.1, 95% CI 3.0 to 87.7) and modified SOFA score ≤4 (adjusted OR 5.1, 95% CI 1.0 to 25.0) as statistically significant risk factors for ARC. The r^2 ^value was 0.59, and the Hosmer-Lemeshow statistic had a significance value of *P *= 0.834, suggesting acceptable goodness of fit. There was no improvement in model performance when continuous variables were utilized.

**Figure 4:**
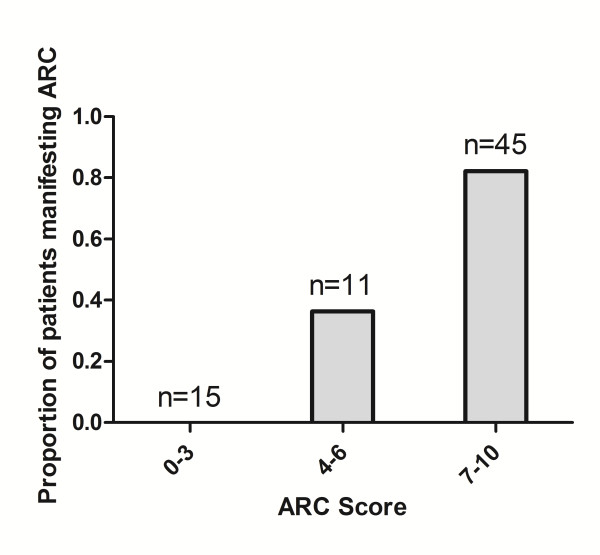
**Proportion of patients manifesting augmented renal clearance with increasing ARC risk scores**. Summated risk scores were grouped into three categories (0 to 3, 4 to 6, 7 to 10) and the proportion of patients manifesting augmented renal clearance determined in each. Higher scores were strongly associated with a greater prevalence of augmented renal clearance (*P* <0.001).

**Figure 5:**
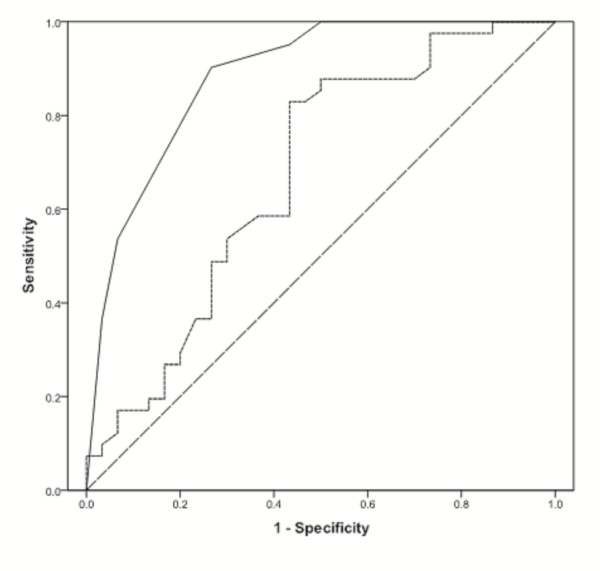
**Receiver Operating Characteristic (ROC) curve of CI (L/min/m^2^) and ARC risk score in predicting augmented renal clearance**. ROC curve of cardiac index, L/min/m^2 ^(dashed line) and ARC risk score (solid line). Cardiac index demonstrates an AUC of 0.67 (95% CI 0.54 to 0.81, *P *= 0.013), whereas the ARC risk score has improved accuracy, with an AUC of 0.89 (95% CI 0.80 to 0.97, *P* <0.001). A diagonal reference line (AUC = 0.5) is also provided.

To further illustrate the relative significance of these covariates, a weighted scoring system was constructed based on the adjusted ORs and their proportions to each other. Age ≤50 years was assigned six points, admission post-trauma three points and modified SOFA score ≤4 one point. Scores were then summated for each patient, with higher totals strongly associated (*P* <0.001) with ARC (see Figure 4). This model was also compared with CI measurement as a predictor of ARC status using ROC analysis (see Figure 5). CI values alone demonstrate an area under the curve (AUC) of 0.67 (95% CI 0.54 to 0.81, *P *= 0.013), whereas the combined ARC score has improved accuracy, with an AUC of 0.89 (95% CI 0.80 to 0.97, *P* <0.001). Separate ROC curves were also constructed utilizing CI values in each diagnostic sub-group (figures not displayed). In those manifesting trauma, CI was less discriminating, with an AUC of 0.57 (95% CI 0.31 to 084, *P *= 0.646), although this variable performed better in sepsis, AUC 0.72 (95% CI 0.57 to 0.87, *P *= 0.015).

## Discussion

This pilot investigation, in the context of a larger study examining β-lactam antibacterial PK in the critically ill [29], has explored the relationship between CI and CL_CR _in a cohort of septic and traumatized patients with normal plasma creatinine concentrations. Overall, ARC was present in more than 50%, similar to a previous report in critically ill patients receiving anti-infective therapy [31]. A greater prevalence of ARC was noted in those suffering multi-trauma (85.7%). In univariate analysis, a statistically significant association between higher CI and ARC (*P *= 0.013) was observed, while in multivariate modelling, age (≤50 years), diagnostic category (trauma) and modified SOFA score (≤4) were identified as significant risk factors for ARC.

These findings principally suggest that the underlying disease process and physiological reserve, more than any specific cardiovascular parameter, are implicated in the development of ARC. This is highly clinically relevant, given the potential for significant sub-therapeutic drug exposure when employing 'standard' doses in such patients. Relevant examples include increased clinical failure [32] or drug resistance [33] with β-lactam antibacterial therapy or sub-optimal venous thromboembolism prophylaxis in those receiving low molecular weight heparin [34].

Multi-trauma has already been identified as a significant risk factor for ARC [9,12,14] and this is further confirmed by our findings. The absence of any correlation between CI and CL_CR _in trauma is likely related to the higher CL_CR _measures observed in this group, the narrow range of recorded cardiac indices (see Figure 1) and the smaller sample size. Furthermore, recruitment of renal reserve [35], typically seen in states characterized by protein loading [36], may potentially augment glomerular filtration in this setting, independent of changes in CI.

Importantly, the high prevalence of ARC observed in the trauma sub-group, despite the limited value of CI measurement as a discretionary variable, has considerable potential ramifications for both future study design [6] and daily prescribing practice. Specifically, this finding reminds the clinician that a 'one size fits all' approach to drug dosing in critical illness, is flawed and requires adjustment for a number of variables, least of which is diagnostic category. The recent poor results from clinical trials of emerging antibacterial agents in ventilator associated pneumonia [6] further illustrate this concept. Selecting a single dosing regimen for all study participants is unlikely to accommodate the range of clinical and physiological characteristics encountered.

The lower prevalence of ARC (39.5%) and greater variability in CL_CR _and CI in the septic sub-group (Figure 1) reflects the heterogeneity of this syndrome and the wider spectrum of age and underlying co-morbid disease. Such variables significantly impact the available physiological reserve and, as such, the likelihood of manifesting augmented clearances. This is evidenced by the strong overall association between ARC, lower modified SOFA scores and age, findings which are consistent with previous literature [37]. Identification of additional drivers of ARC in septic patients is not possible with the current dataset, although this is likely to reflect the interaction between the innate inflammatory response and available organ reserve.

Previous data examining the relationship between CI and renal solute elimination in critical illness are limited. Specifically, Brown *et al*. sequentially assessed CL_CR _in fifty relatively young critically ill post-operative trauma and non-trauma patients while simultaneously measuring CI via a pulmonary artery catheter (PAC) [15]. After exclusion of those receiving inotropes or diuretics and those with sepsis or renal failure, a modest correlation was established between CI and CL_CR _(r = 0.63, *P* <0.01) [15]. Our study extends these findings, with data distinct from a peri-operative setting and suggests a modest correlation between CI and CL_CR _in critically ill septic patients (r = 0.508, *P *= 0.001).

The influence of common critical care interventions on cardiovascular and renal function remains to be accurately determined. Specifically, although improvements in CL_CR _following intravenous fluid administration [25,38] and use of vasopressor agents [26,39] have been noted in large animal models, we did not observe any statistically significant difference in either the requirement for vasopressors (*P *= 0.173) or 24-hour fluid balance (*P *= 0.237) in those manifesting ARC. Importantly, these data could be misleading, as they represent information obtained around the time of drug dosing only and, therefore, fail to consider any prior interventions.

Minimally invasive pulse contour cardiac output analysis was employed in this study primarily due to ease of application and decreasing use of PACs in routine clinical practice [40]. Although mixed results have been reported in prior validation studies [41], particularly with the earlier software [42], later iterations have improved the accuracy of the device [43], with an acceptable percentage error [41] and concordance rate [44] in comparison to PAC thermodilution. Aortic valve abnormalities are still likely to cause discrepancy [45] through distortion of the pulse contour, although they were not actively screened for in our analysis. Importantly, despite the growing use of pulse contour cardiac output analysis in clinical practice, its use in general intensive care remains controversial [46] and must be recognized as a limiting factor in this analysis.

Despite the perceived inaccuracies of any specific device(s), our findings indicate a potential new, unique, direction for minimally invasive CI monitoring in critically ill septic patients. The modest correlation observed between CI and CL_CR_, in addition to the ROC analysis, suggests that elevated values may be viewed as a clinical 'trigger' in patients without AKI, to re-consider the dosing strategy in use, particularly in relation to antibacterial therapy. While additional prospective studies utilizing drug PK data are urgently required, clinical trials examining the efficacy of new agents in this setting must be cognisant of these findings [6]. Importantly, our data is limited temporally, such that we do not report changes in CI and CL_CR _during the ICU stay. As critical illness represents a highly dynamic state, ongoing CI measurement may be even more useful in tailoring drug prescription over time.

We have not included specific drug PK data in these analyses for the following reasons: a) routine measurement of drug levels (β-lactam or otherwise) is infrequent; b) CI and CL_CR _assessment are much more accessible in clinical practice; and c) CL_CR _(allowing identification of ARC) was the primary end-point of interest. In addition, ARC may influence drug handling for many different pharmaceuticals, as CL_CR _is recognized as a key PK covariate for renally eliminated agents [8,16,17]. It is acknowledged that CL_CR _is not a 'gold standard' measure of glomerular filtration (such as inulin clearance), albeit tubular creatinine secretion is unlikely to influence the result at higher filtration rates [47]. Two-hour urinary collections were employed, as prior research has reported acceptable accuracy compared with longer time-periods [48]. The implications of the proposed ARC scoring system are also acknowledged, with the current findings being primarily speculative. Separate, large, multicenter validation studies are required, in order to establish its external validity, and assess any potential clinical utility.

## Conclusions

To our knowledge, this is the first study to correlate CI and CL_CR _in a range of critically ill patients, in addition to investigating the application of pulse contour cardiac output monitoring as a means of identifying augmented renal solute elimination. Our findings suggest that diagnostic category, illness severity, age and organ function are likely to significantly influence the probability of developing ARC and should be more regularly considered in future study design and daily prescribing practice. Specifically, these factors may be useful in identifying patients at risk of altered drug handling in critical illness. While additional PK data are required, these results provide a robust basis on which to undertake larger clinical investigation, specifically focusing on the development of improved drug dosing algorithms in the critically ill.

## Key messages

• Augmented renal clearance appears to be common in critically ill patients with normal plasma creatinine concentrations who are receiving antibacterial therapy in the ICU.

• Young trauma patients, without significant organ dysfunction, appear to be at greater risk.

• The correlation between cardiac index and urinary creatinine clearance is better in critically ill septic patients than in trauma patients.

• Minimally invasive pulse counter cardiac output monitoring may have a role in identifying critically ill septic patients with augmented renal clearance.

## Abbreviations

AKI: acute kidney Injury; APACHE: Acute Physiology and Chronic Health Evaluation; ARC: augmented renal clearance; AUC: area under the curve; BSA: body surface area; CI: cardiac index; CL: clearance; CL_CR_: creatinine clearance; OR: odds ratio; PAC: pulmonary artery catheter; PK: pharmacokinetics; ROC: receiver operator characteristic; SIRS: systemic inflammatory response syndrome; SOFA: Sequential Organ Failure Assessment.

## Competing interests

JL is a consultant to Astra Zeneca and Janssen-Cilag, and has received honoraria from Astra Zeneca, Janssen-Cilag and Wyeth Australia. JR has previously consulted for Janssen-Cilag, Astra-Zeneca, Pfizer and Gilead; has been involved in advisory boards for Janssen-Cilag and Astra-Zeneca; and has received unrestricted grants from Janssen-Cilag, Astra-Zeneca and Novartis. AS has acted as a speaker for, consulted to, or received grant support from: Astellas, Bayer, Forrest, Pfizer, Theravance, and Trius. AU and RB declare they have no conflicts of interest. Astra Zeneca and Edwards Lifesciences provide an annual un-restricted donation to the Burns, Trauma and Critical Care Research Centre (BTCCRC), University of Queensland.

## Authors' contributions

JR and JL conceived the study. JR, AU, RB and JL were involved in protocol development, ethics approval and implementation. AU and JR collected the data. AU, AS, and RB undertook the statistical analysis. AU wrote the initial draft, with all of the remaining authors contributing to subsequent revisions. AU takes responsibility for archiving the data and guarantees the integrity of the paper from inception to publication. All of the authors have read and approved the article for publication.
